# Revocable and Fog-Enabled Proxy Re-Encryption Scheme for IoT Environments

**DOI:** 10.3390/s24196290

**Published:** 2024-09-28

**Authors:** Han-Yu Lin, Pei-Ru Chen

**Affiliations:** Department of Computer Science and Engineering, National Taiwan Ocean University, Keelung 202, Taiwan; 11057032@mail.ntou.edu.tw

**Keywords:** fog computing, proxy, re-encryption, revocation, IoT

## Abstract

As technology advances rapidly, a diverse array of Internet of Things (IoT) devices finds widespread application across numerous fields. The intelligent nature of these devices not only gives people more convenience, but also introduces new challenges especially in security when transmitting data in fog-based cloud environments. In fog computing environments, data need to be transmitted across multiple devices, increasing the risk of data being intercepted or tampered with during transmission. To securely share cloud ciphertexts, an alleged proxy re-encryption approach is a commonly adopted solution. Without decrypting the original ciphertext, such a mechanism permits a ciphertext intended for user A to be easily converted into the one intended for user B. However, to revoke the decryption privilege of data users usually relies on the system authority to maintain a user revocation list which inevitably increases the storage space. In this research, the authors come up with a fog-based proxy re-encryption system with revocable identity. Without maintaining the traditional user revocation list, the proposed scheme introduces a time-updated key mechanism. The time-update key could be viewed as a partial private key and should be renewed with different time periods. A revoked user is unable to obtain the renewed time-update key and hence cannot share or decrypt cloud ciphertexts. We formally demonstrate that the introduced scheme satisfies the security of indistinguishability against adaptively chosen identity and chosen plaintext attacks (IND-PrID-CPA) assuming the hardness of the Decisional Bilinear Diffie–Hellman (DBDH) problem in the random oracle model. Furthermore, compared with similar systems, the proposed one also has lower computational complexity as a whole.

## 1. Introduction

Cloud computing [1] is a technology that provides computing resources and services over the Internet. It allows users to access and share computing resources via the Internet without the need to own, manage, or maintain physical hardware and software. Numerous businesses have begun venturing into the cloud computing sector attracted by its benefits like cost-effectiveness and enhanced productivity. Consequently, cloud services have progressively become integral to our daily lives. However, with the emergence of the IoT [2,3,4], many applications require real-time processing capabilities. For example, in smart transportation systems, real-time feedback of vehicle-driving data collected by sensors is necessary for immediate response to emergencies. Therefore, reducing data transmission time and improving efficiency have become major challenges, which further drives the development of fog computing [5,6,7].

Fog computing is often regarded as an expansion of cloud computing. It emphasizes executing computing at the data source to reduce reliance on central cloud resources, meeting the demands of applications like the IoT. In the IoT environment of a fog computing architecture, fog nodes (FNs) are the core components, widely distributed between data sources and the cloud. If applied in the healthcare industry, it can be used for monitoring patient data and providing real-time diagnosis. Fog computing can provide multi-layered data processing and flexible configuration according to requirements and applications. Although its computing power is weaker compared to the cloud, users can transfer data from closer fog nodes to the cloud. This not only accelerates the response to user demands but also reduces the computational burden on cloud servers.

**Figure 1:**
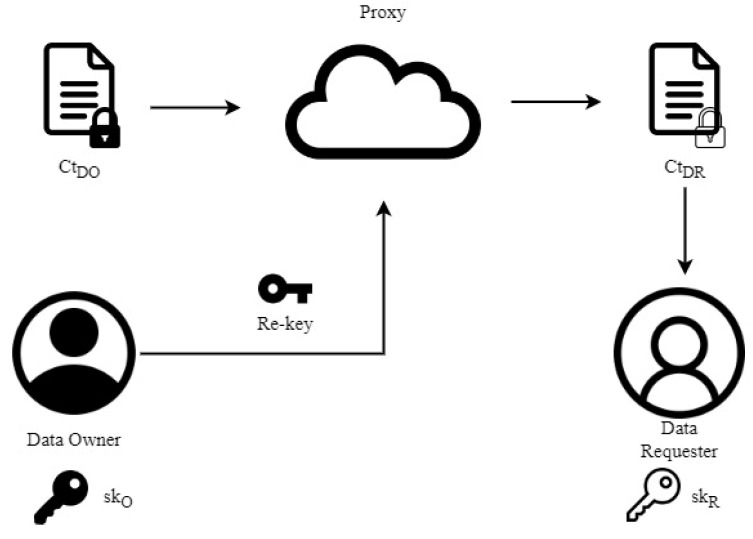
Illustration of re-encryption processes.

Today, the majority of users opt to store their data in cloud storage solutions. When they attempt to share confidential data kept in the cloud with others, they can utilize the proxy re-encryption [8,9,10,11,12] technique introduced by Blaze et al. [13]. The basic concept is illustrated as Figure 1. Specifically, the data owner encrypts data and stores it in the cloud for future use. Since the data is encrypted, the cloud server cannot learn its content without having the data owner’s private key. If the data owner wants to share the data stored in the cloud with a data requester, they can authorize a semi-honest proxy server to perform the re-encryption process. The re-encryption key is generated by the data owner themselves. As the ciphertext is not decrypted during the process, there is no concern about the proxy server gaining information about the plaintext.

### 1.1. Related Works

So far, many proxy re-encryption applications have been proposed, including data sharing, data outsourcing, and cloud data storage. In 2015, Chen et al. [14] addressed the concept of a Verifiable Database with Incremental Updates (Inc-VDB), which enables resource-constrained users to outsource large amounts of data to untrusted servers for retrieval and update. If the server attempts to tamper with the data, the user can detect it. However, when users require frequent and small modifications to the data in the database, they must recalculate new ciphertexts and update the data on the server. For resource-constrained users, such operations are costly. To meet the above application requirements, they proposed a specific scheme for verifiable databases with incremental updates under the CDH assumption.

In 2017, Bankar and Raghatwan [15] proposed an identity-based proxy re-encryption using forward security in a cloud framework. Their system enables the data owner to securely store data in the cloud server by initially encrypting them and sending them to a Trustworthy Third Party (TTP), which further encrypts the data. Additionally, the data owner can manage data storage operations such as upgrades, scaling, and retrieval in the cloud server. Access control mechanisms are implemented by the data owner for the outsourced data. Furthermore, in case of disputes concerning data integrity, the TTP has the authority to determine dishonest users.

Conditional proxy re-encryption [16,17,18] is a frequently used method for applying access control policies to cloud ciphertexts. Nonetheless, the size of the re-encryption keys tends to grow proportionally with the number of condition values, posing challenges for implementation on resource-constrained computational devices. In 2018, Chen et al. [19] introduced a key-aggregate proxy re-encryption scheme designed for secure data sharing in cloud environments. Their research demonstrated that the proposed scheme could effectively facilitate fine-grained access control on files using constant-size re-encryption keys. In 2019, Shen et al. [1] proposed a block design-based key agreement protocol for group data sharing in cloud computing. Their method enables secure key agreement among multiple participants in a cloud environment, reducing the overhead in managing group communications.

In 2020, Zhang et al. [20] addressed a data storage method using identity-based systems and the techniques of proxy re-encryption and fog computing. Identity-based encryption schemes typically have the problem of key escrow since the user private key is issued by the private key generation center (PKG). In their method, even though the PKG has the master private key, it cannot learn the real private key of users. Additionally, the re-encryption keys in their method do not need to be generated through the PKG but can be calculated by the data owner themselves. In 2021, Yao et al. [17] introduced a revocable and identity-based conditional proxy re-encryption scheme tailored for cloud data sharing. This scheme supports ciphertext evolution, meaning that re-encrypted ciphertexts are updated over time, ensuring backward security even if a key is compromised. Yang et al. [21] presented an improved proxy re-encryption scheme with equality testing. They enhanced the functionality by allowing for secure ciphertext comparison without decryption. This improvement is particularly valuable in scenarios requiring secure data matching or deduplication in cloud environments, while maintaining strong security guarantees. Zhang and Li [22] introduced a blockchain-based attribute proxy re-encryption scheme specifically designed for secure medical data sharing. By integrating blockchain technology with attribute-based encryption, the scheme ensures that medical records can be shared securely and transparently, with immutable audit trails.

In 2022, Ge et al. [23] proposed a verifiable and fair attribute-based proxy re-encryption scheme designed for secure and efficient data sharing in cloud environments. The scheme ensures that the re-encryption process is verifiable and maintains fairness, which is critical for ensuring that both data owners and recipients are treated equitably. In the same year, Lin et al. [24] pointed out several security flaws in Zhang et al.’s work [20] and presented a secure variant. They also further proposed a more efficient mechanism in [25]. Nevertheless, in these schemes, the PKG has to maintain a revocation list for fulfilling the functionality of user revocation, which would increase additional storage cost. Motivated by this concern, we will propose a fog-based proxy re-encryption system with revocable identity. In particular, the proposed system utilizes a time-update key algorithm to achieve the user revocation without maintaining a user revocation list.

### 1.2. Motivation and Contributions

In prior revocable schemes [20,24,25], the PKG must maintain a revocation list to manage user revocation, which introduces significant storage overhead as the list grows with each revoked user. This added burden is especially detrimental in resource-constrained environments. Motivated by this limitation, we propose a fog-based proxy re-encryption system that leverages a time-update key algorithm to achieve efficient user revocation without the need for maintaining a traditional revocation list. Our approach offers a more streamlined solution by periodically updating user keys based on time intervals, ensuring that revoked users lose access without requiring constant list maintenance. The proposed system not only simplifies key management but also improves performance metrics. Compared with existing schemes, our method significantly reduces computational overhead by eliminating the need for real-time revocation list queries and updates. Additionally, the time-update key mechanism inherently reduces storage costs and computational complexity, making it more scalable for IoT environments. The findings show that our system achieves the same or better security guarantees, particularly against adaptively chosen identity and chosen plaintext attacks (IND-PrID-CPA), while maintaining lower computational and storage costs. This efficiency makes our scheme highly suitable for fog-enabled IoT applications where resources are limited, and real-time data processing is essential.

The remainder of this paper is organized as follows: Section 2 covers the essential computational assumptions. Section 3 details the proposed scheme. In Section 4, we discuss potential security challenges, provide formal security proof and evaluate performance. Finally, Section 5 concludes the paper and discusses future research directions.

## 2. Preliminaries

This section primarily elucidates the background knowledge to be used in this paper.

### 2.1. Bilinear Pairing

Bilinear pairing is a cryptographic primitive with numerous applications in modern security protocols, especially in identity-based encryption (IBE), attribute-based encryption (ABE), and proxy re-encryption schemes. In identity-based encryption, bilinear pairing allows a trusted authority to generate private keys for users based on their identities, simplifying public key infrastructure management. Pairing-based cryptography is also widely used in short digital signatures, where the efficiency of the pairing operation makes it ideal for environments with limited bandwidth or storage. Another key application is in group key exchange protocols, where bilinear pairing is used to establish secure group communication efficiently. In proxy re-encryption schemes, such as the one proposed in this paper, bilinear pairing enables secure re-encryption without exposing the plaintext or compromising the system’s security guarantees. The definition of bilinear pairing is as follows. Let ***G***_1_ and ***G***_2_ be multiplicative groups of prime order *p*, and *g* an arbitrary generator of ***G***_1_ that is not the identity element. If the following properties hold, then *e*: ***G***_1_ × ***G***_1_ → ***G***_2_ can be regarded as a symmetric bilinear map:

(i) ***Bilinearity:***

If *g* ∈ ***G***_1_ and *i*, *j* ∈ Z_p_^*^, then *e*(*g^i^*, *g^j^*) = *e*(*g*, *g*)*^ij^*.

(ii) ***Non-degeneracy:***

The generator *g* ∈ ***G***_1_ satisfies that *e*(*g*, *g*) ≠ 1.

(iii) ***Computability:***

If *P*, *Q* ∈ ***G***_1_ and there is a polynomial-time algorithm that can efficiently compute *e*(*P*, *Q*), it satisfies computability.

### 2.2. One-Way Hash Function

One-way hash functions are fundamental in many cryptographic protocols, owing to their properties of irreversibility and collision resistance. These functions are extensively used in digital signatures to ensure data integrity and authenticity, where a message is hashed before signing, allowing for efficient verification without revealing the original message. Another common application is password protection: rather than storing passwords directly, systems store hash values of passwords, making it difficult for attackers to recover the original password, even if they gain access to the hashed values. One-way hash functions are also critical in constructing message authentication codes (MACs) and hash-based digital signatures, where they serve to verify data integrity and authentication. Additionally, in blockchain technology, hash functions ensure that data blocks are cryptographically linked, securing the integrity of the chain. A secure one-way hash function should satisfy the following characteristics:

(i) ***Irreversibility (One-way):***

Difficult to analyze in reverse, i.e., impossible to derive the original input from the hash value.

(ii) ***Fixed-length output:***

Any length of message can result in a hash value of the same length.

(iii) ***Fast computation:***

Even with large amounts of data, the hash function can compute within a reasonable time.

(iv) ***Collision resistance:***

Two different inputs producing the same hash value, known as hash collisions, should be extremely rare for a secure hash function.

(v) ***Avalanche effect:***

Slight changes in input values should result in significant changes in the hash value.

- ***Decisional Bilinear Diffie–Hellman (DBDH) Problem***

Given a set of values (*g*, *g^x^*, *g^y^*, *g^z^*, *e*(*g*, *g*)*^xyz^*, *γ*) in which *g*, *g^x^*, *g^y^*, *g^z^* ∈ ***G***_1_^4^ and *e*(*g*, *g*)*^xyz^*, *γ* ∈ ***G***_2_^2^, the DBDH problem is to decide whether the equality *e*(*g*, *g*)*^xyz^* = *γ* holds or not.

- ***Decisional Bilinear Diffie–Hellman (DBDH) Assumption***

When the advantage of any probabilistic polynomial-time adversary in solving the DBDH problem is negligible, the DBDH assumption holds.

## 3. Proposed IB-PRE-FCAK Scheme

This section will introduce the proposed method and the definition of each algorithm.

### 3.1. Algorithms Definition

Here are the definitions of the algorithms used in the proposed system:

- **System Initialization (SI):** Given the security parameter *τ*, it generates the public parameters (*PP*), the master secret key (*Msk*) and the master public key (*Mpk*) required for the system
- **Initial Private Key Generation (IPKG):** Given the public parameters (*PP*), the master private key (*Msk*), and the identity of the user to be registered (*ID_u_*), it generates the initial private key (*ipk_u_*) for the user.
- **Time-Update Key Generation (TUKG):** Given the public parameters (*PP*), the master private key (*Msk*), the identity of the user to be registered (*ID_u_*), and the time period (*n*), it generates the time-update key (*tuk_u_*_, *n*_) for the user.
- **Encryption:** Given the parameters (*PP*), the time period (*n*), a symmetric key (*Y*), a plaintext (*m*), and the identity of data owner (*ID_O_*), it generates the encrypted ciphertext (*CT*).
- **Query:** Given the identity of the data requester (*ID_R_*) and the file index (*Fi*) to be requested, it generates the corresponding query token (*Θ*).
- **Re-encryption Key Generation (RenKG):** Given the parameters (*PP*), the query token (*Θ*), and data owner’s private key (*sk_O_*_, *n*_), it generates the re-encryption key (*renk_O_*_, *n*_).
- **Re-encryption:** Given the parameters (*PP*), the *ID* of the data requester (*ID_R_*), the re-encryption key (*renk_O_*_, *n*_) sent by the data owner, the file index (*Fi*), the identity of the data owner (*ID_O_*), and an original ciphertext (*CT*), it calculates the re-encrypted ciphertext (*RCT*).
- **Decryption:** The decryption algorithm can be divided into two types: one is for the data owner to decrypt and the other is for the data requester to decrypt. Specifically, given the public parameters (*PP*), a user private key (*sk_O_*_, *n*_ or *sk_R_*_, *n*_), and the ciphertext (*CT*) or the re-encrypted ciphertext (*RCT*), it calculates the symmetric key (*Y*) for deriving the original plaintext *m*.

### 3.2. Method Construction

**Figure 2:**
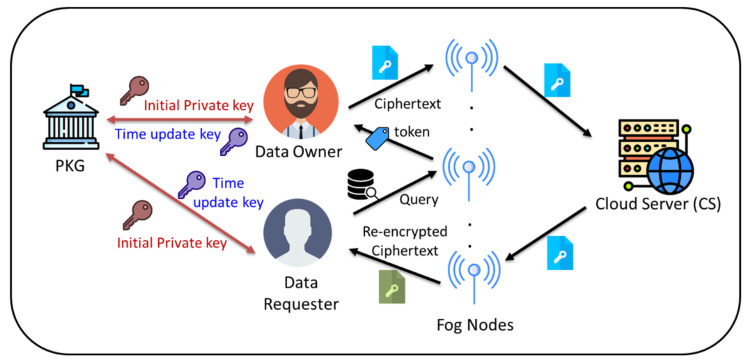
Illustration of the proposed system model.

This section describes the proposed method and architecture. Figure 2 depicts the system model and participating parties in the proposed scheme.

- System Initialization (SI(1*^τ^*))

The Private Key Generation Center (PKG) takes the security parameter *τ* as input and generates the public parameters *PP*, the master private key *Msk* and the master public key *Mpk*. Let *PP* = {$e,G_{1},G_{2},g,{p,\;H}_{1},H_{2},H_{3},Mpk,\left(SymE,SymD\right)$}, where the symbols represent the following:

1. *G*_1_ and ***G***_2_ are two prime-order multiplicative groups of the order *p*, g is a generator of ***G***_1_ and *e* is a bilinear mapping function, i.e., *e*: ***G***_1_ × ***G***_1_ → ***G***_2_.

2. *H*_1_, *H*_2_ and *H*_3_ are three collision-resistant hash functions, i.e., *H*_1_: {0, 1}* → ***G***_1_, *H*_2_: {0, 1}* → ***G***_1_ and *H*_3_: ***G***_2_ → ***G***_1_.
3. *Msk* is defined as ψ arbitrarily selected from Z_p_^*^ and the *Mpk* is calculated as $g_{1}=g^{\psi }$.
4. $\left(SymE,SymD\right)$ are symmetric encryption and decryption functions, respectively.

- Initial Private Key Generation (IPKG(*PP*, *Msk*, *ID_u_*))

The steps for generating the initial private key are as follows:

User ${ID}_{u}$ sends his/her identity ${(ID}_{u})$ to the PKG;

1. PKG computes the initial private key ${ipk}_{u}={H_{2}({ID}_{u})}^{\psi }$ and returns it to the user ${ID}_{u}$;
2. The accuracy of the initial private key can be checked with the equation
  $$e\left({ipk}_{u},g\right)=e(H_{2}({ID}_{u}),g_{1}).$$

- Time-Update Key Generation (TUKG(*PP*, *n*, *Msk*, *ID_u_*))

The steps for generating the time-update key associated with the time period *n* are as follows:

1. User ${ID}_{u}$ sends ${(ID}_{u},n)$ to the PKG;
2. PKG computes the time-update key ${tuk}_{u,\;n}={H_{1}({ID}_{u}∥n)}^{\psi }$ and returns it to the user ${ID}_{u}$;
3. The accuracy of the key can be checked with the equation
  $$e\left({tuk}_{u,n},g\right)=e(H_{2}({ID}_{u}∥n),g_{1});$$
4. User ${ID}_{u}$ can compute the complete private key by using the previously obtained ${ipk}_{u}$ as
  (1) $${sk}_{u,n}={ipk}_{u}\cdot {tuk}_{u,n}={\left(H_{1}({ID}_{u}∥n)H_{2}({ID}_{u})\right)}^{\psi }$$

- Encryption(*PP*, *n*, *Y*, *m*, *ID_O_*)

Data owner ${ID}_{O}$ inputs public parameters *PP*, a time period *n*, a symmetric key *Y*, a file index *Fi*, a plaintext $m=\left(m_{1},m_{2},\ldots ,m_{w}\right)$ along with his/her identity ${ID}_{O}$, and then selects a random number *l* ∈ Z_p_^*^ to compute the ciphertext *CT* = {*CT*_0_ = (*C*_1_, *C*_2_), *CT*_1_} as

(2) $$C_{1}=Y\cdot e(g_{1},{\left(H_{1}({ID}_{O}∥n)H_{2}({ID}_{O})\right)}^{l})$$

(3) $$C_{2}=g^{l}$$

(4) $${CT}_{1}=(SymE(Y,m_{1}),\ldots ,SymE(Y,m_{w}))$$

After computation, ${ID}_{O}$ sends $\left(n,\;CT,{ID}_{O},Fi\right)$ to the nearby fog nodes which will store partial ciphertext $\left({CT}_{0},{ID}_{O},Fi\right)$ and forward the other partial ciphertext $\left({CT}_{1},{ID}_{O},Fi\right)$ to the cloud server.

- Query(*PP*, *Fi*, *ID_R_*)

To request the cloud ciphertext associated with the file index *Fi*, a data requester ${ID}_{R}$ selects a random number ϱ ∈ Z_p_^*^, computes

(5) $$Q=g^{\varrho }$$

and sends $\left(Fi,{ID}_{R},Q\right)$ to nearby fog nodes which will generate the corresponding query token $\Theta =(Q,{ID}_{R})$ and deliver it to the data owner ${ID}_{O}$.

- Re-encryption Key Generation (RenKG(*PP*, *Θ*, *sk_O_*_, *n*_))

First, data owner ${ID}_{O}$ selects random numbers *c*, *v* ∈ Z_p_^*^ and calculates the following values forming the re-encryption key:

(6) $${rk}_{1}={g_{1}}^{c}$$

(7) $${rk}_{2}=({sk}_{O,n}){rk}_{1}/H_{3}(e\left(H_{2}({ID}_{R})Q^{\upsilon },g_{1}\right))$$

(8) $${rk}_{3}=e(g^{\upsilon },g_{1})$$

After computation, the re-encryption key ${renk}_{O,\;n}=\left({rk}_{1},\;{rk}_{2},\;{rk}_{3}\right)$ is sent to fog nodes.

- Re-encryption(*PP*, *ID_R_*, *renK_O_*_, *n*_, *Fi*, *ID_O_*, *CT*)

After receiving *renk_O_*_, *n*_, fog nodes perform re-encryption calculations as:

(9) $${RC}_{1}=C_{1}\cdot e\left({rk}_{1},C_{2}\right)$$

(10) $${RC}_{2}=C_{2}$$

(11) $${RC}_{3}={rk}_{2}$$

(12) $${RC}_{4}={rk}_{3}$$

Then the identity of the data owner and the file index $\left({ID}_{O},\;Fi\right)$ are transmitted to the cloud server which will return the stored partial ciphertext ${CT}_{1}$. Fog nodes then send the re-encrypted ciphertext $RCT=\left({RCT}_{0},{CT}_{1}\right)$ to the data requester ${ID}_{R}$, where ${RCT}_{0}=({RC}_{1},\;{RC}_{2},\;{RC}_{3},\;{RC}_{4})$.

- Decryption(*CT*, *sk_u_*_, *n*_)

The decryption processes can be divided into two cases:

1. The data owner ${ID}_{O}$ decrypts the ciphertext *CT* by calculating the symmetric key *Y* as:
  (13) $$Y=\frac{C_{1}}{e(C_{2},\;{sk}_{O,\;n})}$$
  (14) $${\left\{m_{i}=SymD\left(Y,\;SymE\left(Y,m_{i}\right)\right)\right\}}_{i=1,\;\ldots ,\;w}$$
  The correctness of *Y* is derived as follows:
  $$Y=\frac{C_{1}}{e\left(C_{2},\;{sk}_{O,\;n}\right)}=\frac{Y\cdot e(g_{1},\;(H_{1}\left({ID}_{O}∥n\right){H_{2}({ID}_{O}))}^{l})}{e(g^{l},{\left(H_{1}\left({ID}_{O}∥n\right)H_{2}\left({ID}_{O}\right)\right)}^{\psi })}=Y$$
2. The data requester ${ID}_{R}$

decrypts the re-encrypted ciphertext *RCT* by computing the meta parameter *E* and the symmetric key *Y* as:

(15) $$E={RC}_{3}\cdot H_{3}(\frac{{{RC}_{4}}^{\varrho }\cdot e({sk}_{R,\;n},\;g)}{e(H_{1}({ID}_{R}∥n),\;{\;g}_{1})})$$

(16) $$Y=\frac{{RC}_{1}}{e\left(E,\;{\;RC}_{2}\right)}$$

(17) $${\left\{m_{i}=SymD\left(Y,\;SymE\left(Y,\;m_{i}\right)\right)\right\}}_{i=1,\;\ldots ,\;n}$$

The correctness of *Y* is derived as follows:

$$Y=\frac{{RC}_{1}}{e\left(E,\;{RC}_{2}\right)}=\frac{Y\cdot e(g_{1},\;{{{(H}_{1}\left({ID}_{O}∥n\right)H}_{2}\left({ID}_{O}\right))}^{l})e\left(g^{lc},\;g_{1}\right)}{{e({(H}_{1}\left({ID}_{O}∥n\right)H_{2}\left({ID}_{O}\right))}^{\psi }{g_{1}}^{c},\;g^{l})}=Y$$

## 4. Security Proof and Comparison

This section will utilize the DBDH hardness assumption to conduct a security proof of the scheme proposed in this paper.

### 4.1. Security Analysis and Proof

Based on the proposed scheme, we first address some potential solutions to the challenges related to reliability and security in revocable and fog-enabled proxy re-encryption schemes for IoT environments:

1. **Time Synchronization Issues:** The scheme uses time-updated keys for user revocation. A solution to time synchronization issues is to introduce a tolerance window for time discrepancies across fog nodes and IoT devices. This allows nodes to remain synchronized within acceptable margins and can be further enhanced by employing distributed time synchronization protocols like the Network Time Protocol (NTP).
2. **Increased Computational Overhead:** While proxy re-encryption can increase computational overhead, optimizing the encryption algorithm for lightweight IoT devices is essential. A potential approach is using hardware-accelerated cryptography or offloading heavy computations to fog nodes, reducing the burden on resource-constrained devices.
3. **Limited Key Lifespan:** The scheme introduces time-updated keys, which must be periodically regenerated. To address the risk of frequent key renewal, extending key lifespan through efficient key management policies or using predictive analytics to minimize renewal intervals based on activity patterns can reduce the key-update overhead.
4. **Vulnerability to Replay Attacks:** The system could mitigate replay attacks by introducing a nonce (a unique value) in each transaction, making each message or data exchange unique. This approach ensures that even if an attacker intercepts and resends a message, it will be rejected as the nonce has already been used.
5. **Increased Maintenance Requirements:** Automating key management and revocation processes through smart contracts or decentralized identity management systems can help reduce the maintenance burden. These systems can track key usage, revocation, and renewal with minimal manual intervention.
6. **Risk of Key Exposure:** To prevent key exposure, multi-factor authentication (MFA) and the use of hardware security modules (HSMs) to store sensitive keys can provide additional layers of protection. Additionally, key-splitting techniques, such as Shamir’s Secret Sharing, can distribute key fragments across multiple fog nodes, ensuring that exposure of one fragment does not compromise the entire key.

In the following parts, we will prove that the scheme proposed in this paper satisfies the security of indistinguishability against adaptively chosen identity and chosen plaintext attacks (IND-PrID-CPA) in the random oracle model.

**Definition 1. **  ***(IND-PrID-CPA)*** *If in the following game, no probabilistic polynomial-time (PPT) adversary* A *can defeat the challenger* B *with a non-negligible advantage, it means that the scheme proposed in this paper achieves the security of indistinguishability against adaptively chosen identity and chosen plaintext attacks (IND-PrID-CPA):*

***Setup:*** *Initially, the challenger* B *executes the algorithm of SI*$\left(1^{\lambda }\right)$ *to generate PP and Msk. Then he/she sends PP to* A.

***Phase 1:*** A *adaptively performs the queries below:*

- *IPKG Query:*

*The adversary* A *selects a user and sends his/her ID to* B *who runs the IPKG(PP, Msk, ID) algorithm to compute and obtain the private key (*${ipk}_{ID}$*), and then submits it to* A.

- *TUKG Query:*

A *selects a user and sends his/her ID and the time period n to* B *who runs the TUKG(PP, n, Msk, ID) algorithm to compute and obtain the corresponding time-update key (*${tuk}_{ID,\;n}$*), and then returns it to* A.

- *RenKG Query:*

*The adversary* A *selects two legitimate users and sends their IDs (say ID_O_ and ID_R_) along with the desired file index Fi and the time period n to* B*. Then* B *invokes the IPKG and the TUKG algorithms to obtain the private key* ${sk}_{{ID}_{O,\;n}}$*. Finally,* B *executes the Query(PP, Fi, ID_R_) algorithm to generate a token Θ and returns the result of the RenKG(PP, Θ, sk_O, n_) algorithm to* A.

***Challenge:*** A *defines the aimed user identity* ${ID}^{*}$, the data $m^{*}=\left({m_{1}}^{*},{\;m_{2}}^{*},\ldots ,{{\;m}_{w}}^{*}\right)$, a time period $n^{*}$, and two symmetric keys of equal length $\left(Y_{0},\;{\;Y}_{1}\right)$. B, *using the input* $\left(PP,{\;ID}^{*},{\;m}^{*},{\;n}^{*},Y_{\lambda }\right)$ *where* $\lambda \in_{R}\left\{0,1\right\}$*, generates the ciphertext* ${CT}^{*}$ *and returns it to* A.

***Phase 2:*** A *can execute the queries of Phase 1 upon receiving the challenge, but the relevant restrictions are as follows:*

1. *The IPKG query of the target identity* ${ID}^{*}$ *cannot be made.*

2. *If*A *is already a revoked user (and possesses the initial private key), then no TUKG queries for the target time period* $n^{*}$ *are allowed.*

3. *RenKG queries related to the target identity* $\left({ID}^{*},\;{ID}_{u}\right)$ *or* $\left({ID}_{O},\;{ID}^{*}\right)$ *cannot be executed.*

4. *The number of queries is limited by the maximum execution times of IPKG queries*$q_{ipk}$ *, TUKG queries* $q_{tuk}$*, and RenKG queries* $q_{renK}$.

***Guess:*** *When the adversary* A *finishes Phase 2, the output isa bitλ′. When the conditionλ′=λ, the adversary* A *is the winner of the game. Therefore, the adversary* A *’s advantage, i.e., Adv(*A*), can be denoted asAdv(*A*) = | Pr[λ′=λ]− 1/2 |.*

**Theorem 1.**  *Let* $H_{1}\;and\;H_{2}$ *be random oracles. The proposed mechanism of this paper provides IND-PrID-CPA security under the DBDH assumption. In simple terms, if a PPT adversary* A *can break the IND-PrID-CPA security of the proposed system with a non-negligible advantage ε, within the constraints of maximum query numbers* $q_{ipk}$*,* $q_{tuk}$*, and* $q_{renK}$*, there is an algorithm* B *capable of breaking the DBDH problem with a non-negligible advantage* $\varepsilon^{\prime }$ *in which*

$$\varepsilon \prime \;\text{≥}\;\frac{\varepsilon }{e(q_{ipk}+q_{renK}+1)}$$

*Here, e represents the base of natural logarithm*.

**Proof:**  Let $\left(g,\;g^{f},\;g^{s},\;g^{k},\;{e\left(g,g\right)}^{fsk},\;\gamma \right)$ be an instance of the DBDH problem obtained by B, where f, s, k ∈ Z_p_^*^ and $\gamma \in G_{2}$. B will utilize the advantage of the opponent A to determine whether *γ* is equal to ${e\left(g,\;g\right)}^{fsk}$ or not.

**Setup:** First, B executes the algorithm of SI(1*^λ^*) to generate $PP=\left\{G_{1},\;G_{2},\;e,\;g,\;p,\;Mpk,\;SymE,\;SymD,\;H_{3}\right\}$, where $Mpk=P=g^{s}$ and the corresponding *Msk* is implicitly defined as *s* which is unknown to B. Then *PP* is delivered to A.

**Phase 1:** A adaptively performs the following queries:

- *H*_1_(*ID_i_* || *n*) oracle:

For any query of the form $H_{1}({ID}_{i}|\left|n\right)$, B will use $\left({ID}_{i},n\right)$ as an index to search the *H*_1_-table, denoted as HT1. If no entry is found, B will choose a value *bt*_1_ such that Pr[*bt_1_* = 1] = *ψ* in which the value *ψ* will be decided later. If *bt*_1_ = 0, B calculates ${HO}_{1}={\left(g^{s}\right)}^{s_{1}}$, in which $s_{1}$∈_R_ Z_p_^*^. If *bt_1_* = 1, B calculates ${HO}_{1}=g^{s_{1}}$. Subsequently, HT1 is updated to HT1 ∪ {$\left({ID}_{i},\;n,{bt}_{1},s_{1},{HO}_{1}\right)$}, and *HO*_1_ is returned to A.

- *H*_2_(*ID_i_*) oracle:

For any query of the form $H_{2}({ID}_{i})$, B uses (*IDᵢ*) as an index to search the *H*_2_-table, denoted as HT2. If no entry is found, B computes ${HO}_{2}=g^{s_{2}}$, where $s_{2}$∈_R_ Z_p_^*^. Then, HT2 is updated to HT2 ∪ {$\left({ID}_{i},s_{2},{HO}_{2}\right)$}, and ${HO}_{2}$ is returned to A.

- IPKG Query:

For an IPKG query with *ID_i_*, B utilizes *ID_i_* to be the key value and seeks for a matching entry $\left({ID}_{i},s_{2},{HO}_{2}\right)$ in HT2. If no entry is found, B invokes the *H*_2_(*IDᵢ*) query on behalf of A, computes ${ipk}_{i}=P^{s_{2}}$ and returns it to A.

- TUKG Query:

For a TUKG query with (*ID_i_*, *n*), B utilizes (*ID_i_*, *n*) to be the key value and seeks for a matching entry $\left({ID}_{i},n,{{bt}_{1},\;s}_{1},{HO}_{1}\right)$ in HT1. If no entry is found, B invokes the *H*_1_(*ID_i_*) query on behalf of A. When *bt*_1_ = 0, the process terminates; otherwise, B computes ${tuk}_{i,n}=P^{s_{1}}$ and returns it to A.

- RenKG Query:

For any RenKG query of $\left({ID}_{O},{ID}_{u},\;Fi,\;n\right)$, where ${ID}_{O}\neq {ID}_{u}$ and *ID_u_* has not been revoked, B first derives the complete private key ${(sk}_{O,\;n})$ through the IPKG and the TUKG queries and fetches the associated data kept in HT1. When *bt*_1_ = 0, the process terminates. Otherwise, B selects $\varrho ,\;c,v\in Z_{p}$ to compute $Q=g^{\varrho }$, ${rk}_{1}=P^{c}$, ${rk}_{2}=\frac{{ipk}_{O}\cdot {tuk}_{O,n}\cdot P^{c}}{H_{3}(e(H_{2}\left({ID}_{u}\right)Q^{v},\;P))}$ and ${rk}_{3}=e\left(g^{v},\;P\right)$. Then B returns the re-encryption key ${renk}_{O,\;n}=\left({rk}_{1},\;{rk}_{2},\;{rk}_{3}\right)$ to A.

**Challenge:**A selects an aimed user ${ID}^{*}$, the data $m^{*}=\left({m_{1}}^{*},{m_{2}}^{*},\ldots ,{m_{w}}^{*}\right)$, a time period $n^{*}$, and two symmetric keys of equal length $\left(Y_{0},\;Y_{1}\right)$. B generates the ciphertext ${CT}^{*}=\{n^{*},{{{{CT}_{0}}^{*}=(C}_{1}}^{*},\;{C_{2}}^{*}),\;{{CT}_{1}}^{*}\}$ using the input $\left(PP,{ID}^{*},m^{*},\;n^{*},Y_{\lambda }\right)$ where *λ* ∈ {0,1} with the processes below:

**Step 1** Assume, without loss of generality, that A has already made a query to the hash oracle $H_{1}$ corresponding to ${ID}^{*}$. B aborts when ${{bt}_{1}}^{*}$ = 1.

**Step 2** Otherwise, B fetches the value *s*_1_ from the table HT1 to compute:

$${C_{1}}^{*}=Y_{\lambda }\cdot \gamma^{{s_{1}}^{-1}}\cdot e(P,\;{\left(g^{k}\right)}^{s_{2}})$$

where

$$H_{2}({ID}^{*})=g^{s_{2}}$$

$${C_{2}}^{*}=g^{k}$$

$${{CT}_{1}}^{*}=(SymE(Y_{\lambda },\;{m_{1}}^{*}),\ldots ,\;SymE(Y_{\lambda },\;{m_{w}}^{*}))$$

Finally, B returns the ciphertext ${CT}^{*}=\left(n^{*},{C_{1}}^{*},{C_{2}}^{*},{{CT}_{1}}^{*}\right)$to A.

**Phase 2:** Upon receiving the challenge ciphertext CT^*^, A could continue executing its queries in Phase 1, but must adhere to the relevant constraints defined in Definition 1.

**Guess:** After Phase 2 finishes, A will output a bit *λ*’. When the condition *λ*’ = *λ* holds, B will output 1, indicating that $\gamma {=e(g,\;g)}^{fsk}$; otherwise, it will output 0, indicating that ${\gamma \neq e(g,g)}^{fsk}$.

**Analysis:** According to the challenge stage, if ${e(g,\;g)}^{fsk}=\gamma$, the created ciphertext CT^*^ should be valid, which means that the adversary A has a non-negligible advantage to win the game. We can express that as Adv(A) = | Pr[*λ’* = *λ*] *−* 1/2 | ≥ *ε*. Conversely, on condition that ${\gamma \neq e(g,\;g)}^{fsk}$, meaning that the ciphertext is invalid, we learn that Pr[λ’ = *λ*] = 1/2. Let “Good” be the event of perfect simulation without unintended termination. We thus can represent the advantage of B in solving the DBDH problem to be:

$$|\mathrm{Pr}\left[\left(g,\;{\;g}^{f},\;{\;g}^{s},\;{\;g}^{k},{\;e\left(g,g\right)}^{fsk}\right)=1]-Pr[\left(g,\;g^{f},\;g^{s},\;g^{k},\gamma \right)=1\right]|\geq |\left(\frac{1}{2}+\varepsilon \right)-\frac{1}{2}|\cdot \mathrm{Pr}\left[Good\right]=\varepsilon \cdot \mathrm{Pr}\left[Good\right]$$

In order to more accurately estimate Pr[Good], we consider the probabilities of the following events:

E1: All TUKG queries are perfectly performed without termination;

E2: All RenKG queries are perfectly performed without termination;

E3: The challenge stage is perfectly performed without termination.

Seeing that E1, E2 and E3 are all independent events, the probability Pr[Good] could be written as Pr[E1] · Pr[E2] · Pr[E3]. In TUKG queries, B terminates when the ${bt}_{1}$ associated with ID^*^ is 0, which means $Pr[E1]\leq \Psi^{q_{ipk}}$. In RenKG queries, B terminates when the ${bt}_{1}$ associated with *ID_O_* is 0, with a probability of $Pr[E2]\leq \Psi^{q_{renK}}$. Also, in the challenge stage, B terminates when the ${{bt}_{1}}^{*}$ associated with ${ID}^{*}$ is 1, with a probability of Pr[E3] ≤ (1 − *Ψ*). Putting these probability events together, we obtain:

$$\mathrm{Pr}\left[Good\right]\leq {\left(\Psi \right)}^{q_{ipk}}{\left(\Psi \right)}^{q_{renK}}(1-\Psi )={\left(\Psi \right)}^{q_{ipk}+q_{renK}}(1-\Psi )$$

The probability Pr[Good] has the maximum value of $\frac{1}{e(q_{ipk}+q_{pr}+1)}$ in which *e* is the base of a natural logarithm when we set $\Psi =1-\frac{1}{q_{ipk}+q_{renK}+1}$. Consequently, the advantage of B in solving the DBDH problem can be expressed as $\varepsilon \prime \geq \frac{\varepsilon }{e(q_{ipk}+q_{renK}+1)}$. □

### 4.2. Comparison

This subsection will conduct performance analysis and functionality comparisons of similar studies, including Han et al.’s [26] (referred to as HSM), Zhang et al.’s [20] (referred to as ZBW), Lin et al.’s [24] (referred to as LTTC), and Lin et al.’s [25] (referred to as LTTF).

Table 1 summarizes the comparison of functionality and security. From this table, it can be observed that the study of HSM does not support user revocation while other studies, although capable of revoking users, require the PKG to maintain a user revocation list continuously. As the number of revoked users increases over time, the amount of data that needs to be maintained also becomes substantial. The proposed system provides user revocation through time-updated key generation. By combining the current time-update key and the initial private key, users can only use their complete and effective private keys for generating re-encryption keys and decryption. If a user is revoked, the PKG will no longer issue time-updated keys, meaning the revoked user will no longer be able to carry out re-encryption key generation and decryption.

**Figure 3:**
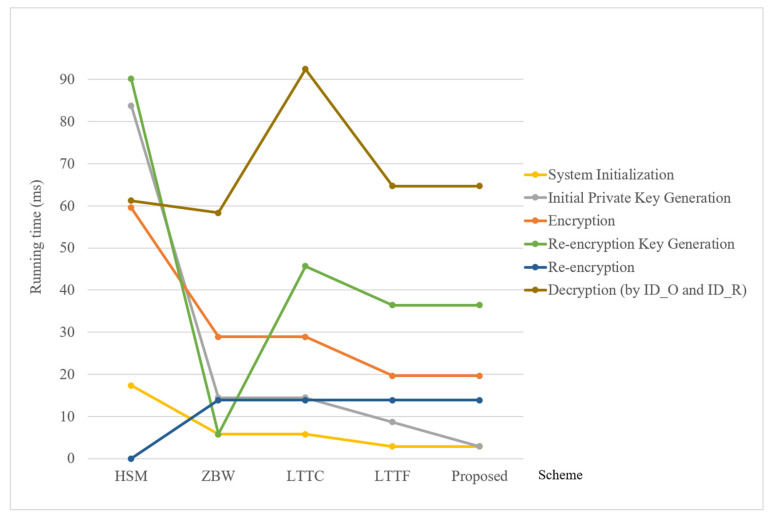
Comparisons of estimated running time.

Table 2 provides a comparison of computational efficiency. We consider the most time-consuming bilinear pairing operation (denoted by “B” in the table) and exponentiation operations (denoted, respectively, by “C” and “D” for computation in ***G***_1_ and ***G***_2_). To provide better experimental results of computation complexity for compared mechanisms, we use Figure 3 to illustrate the estimated running time of each scheme. In particular, according to the study of Zhu et al.’s work [27], one operation of the above “B”, “C” and “D” computation would separately cost 13.864 ms, 2.893 ms and 9.263 ms. From this comparison, we observe that the efficiency of initial private key generation of our system is superior to previous methods. Compared to LTTF, though our scheme includes a newly added time-update key generation algorithm, the overall complexity of our system is reduced. However, the overhead of decryption by data requesters in our system is still higher than that of the HSM and the ZBW schemes. It might be improved by adding a partial decryption process to either the cloud server or the fog node for reducing the computation costs of data requesters in the future.

## 5. Conclusions

Cloud computing has greatly changed the lifestyle of people as well as boosting the development of the Internet of Things. As people require to access more and more online data, the need for real-time processing is also gradually increasing. The emergence of so-called fog computing could off-load the computational burdens of clouds due to its distributed nature and the ability of quick response. In fog-enabled cloud applications, the security of data transmission is crucial. In this research, the authors extend the previous study into a fog-based proxy re-encryption scheme with revocable identity. In the proposed method, the re-encryption key is generated by the data owner, which means that the data owner has absolute control over who has the privilege to access his or her cloud ciphertext. Additionally, the revocation of users is achieved through time-updated keys with time intervals. Specifically, the complete private key is obtained by integrating the initial private key and the time-updated key. The private key is not only for re-encryption key generation, but also for ciphertext decryption. That is to say, when the PKG no longer issues time-updated keys to revoked users, he/she cannot share or retrieve cloud ciphertexts. The proposed scheme also owns the security of IND-PrID-CPA under the assumption of DBDH in random oracle models. Compared to previous research, the proposed method achieves the functionality of user revocation without using the traditional revocation list and the overall computational costs are lower, which makes our system well suited for fog-based cloud environments.

While the proposed fog-based proxy re-encryption scheme with revocable identity offers significant improvements in computational efficiency and eliminates the need for a revocation list, there are some limitations to consider. First, our scheme is not designed to resist the quantum attack which is emerging in cryptographic research. Additionally, although computational overhead is reduced, there is still a burden on resource-constrained IoT devices during encryption and decryption processes, which could be problematic for low-power or low-capacity devices. In future research, we will design an appropriate partial decryption process for the cloud server to reduce the computation overhead of data requesters as well as the communication costs between data requesters and the cloud server. Exploring enhanced security models, such as using fully homomorphic encryption or quantum-resistant algorithms, could also help strengthen the system against advanced attacks.

## Figures and Tables

**Table 1:** Comparisons of functionality and security.

	Scheme	HSM	ZBW	LTTC	LTTF	Proposed
Item						
**Support user revocation**		No	Yes	Yes	Yes	Yes
**Resist revoked user attack**		−	−	Yes	Yes	Yes
**Resist dishonest proxy server**		Yes	No	Yes	Yes	Yes
**Without user revocation list**		−	No	No	No	Yes

**Table 2:** Comparisons of computational cost.

	Scheme	HSM	ZBW	LTTC	LTTF	Proposed
Phase						
**System Initialization**		6C	2C	2C	C	C
**Initial Private Key Generation**		5B + 5C	5C	5C	3C	C
**Time-Update Key Generation**		n.a.	n.a.	n.a.	n.a.	C
**Encryption**		3B + 3C + D	B + 2C + D	B + 2C + D	B + 2C	B + 2C
**Query**		2C	C	C	C	C
**Re-encryption Key Generation**		5B + 4C + D	2C	2B + 3C + D	2B + 3C	2B + 3C
**Re-encryption**		0	B	B	B	B
**Decryption (by *ID_o_*)**		2B	2B	2B	B	B
**Decryption (by *ID_u_*)**		2B + 2C	2B + C	4B + D	3B + D	3B + D

## Data Availability

Data is contained within the article.
